# Oxyresveratrol suppressed melanogenesis, dendrite formation, and melanosome transport in melanocytes via regulation of the MC1R/cAMP/MITF pathway

**DOI:** 10.1038/s41598-025-05248-x

**Published:** 2025-07-01

**Authors:** Jianhua Zhang, Shichao Liu, Wenjiao Guo, Yun Huang, Na Li

**Affiliations:** 1 Guangdong Sensitive Skin Care Engineering Technology Research Center, 544 Guangzhou Avenue North, Guangzhou, 510000 Guangdong China; 2 N.O.D topia (HongKong) Biotechnology Co., Ltd., HongKong, 999077 China; 3 N.O.D topia (GuangZhou) Biotechnology Co., Ltd., 510000 Guangzhou, China; 4 Simpcare (GuangZhou) Biotechnology Co., Ltd., 510000, Guangzhou, China

**Keywords:** Oxyresveratrol, Melanogenesis, Melanosome transport, Human tyrosinase, Melanoma, Melanoma, Kinesin, Small GTPases

## Abstract

Oxyresveratrol, a natural derivative of resveratrol, has been shown to possess antimelanogenic properties. However, the underlying mechanism and its effect on melanin transfer remain poorly understood. In this study, the effects and mechanisms of oxyresveratrol on melanogenesis, dendrite formation, and melanosome transport were investigated. In vitro assays indicated that oxyresveratrol is a potent inhibitor of human tyrosinase, with an IC_50_ value of 2.27 µg/mL. Treatment of B16F10 melanoma cells with oxyresveratrol suppressed melanogenesis through the down-regulation of the MC1R/cAMP/MITF signaling pathway. In a co-culture model of B16F10 and HaCaT cells, oxyresveratrol inhibited both melanin transfer and dendrite formation by down-regulating the expression of small GTPases (CDC42, RAB17, RAB11B, RAC1) and the kinesin KIF5B. These findings suggested that oxyresveratrol may serve as a promising therapeutic agent for pigment-related disorders by inhibiting melanogenesis, dendrite formation, and melanosome transport.

**Supplementary Information:**

The online version contains supplementary material available at 10.1038/s41598-025-05248-x.

## Introduction

Melanin plays a critical role in protecting the skin from ultraviolet radiation (UV); however, excessive production and its abnormal distribution can lead to various dermatological conditions, including melasma, skin aging, and in some cases, skin cancer. Consequently, regulating melanin accumulation is crucial for the prevention and treatment of these conditions.

Melanin is synthesized in the melanosomes of melanocytes and subsequently transferred to and accumulated in keratinocytes. The maturation of melanosome occurs in four stages: stages I and II involve the formation of PMEL (also known as gp100) fibrils, while stages III and IV are associated with melanin synthesis^1,2^. Melanin is produced through the stepwise enzymatic conversion of tyrosine, mediated by tyrosinase (TYR), TRP-1, and TRP-2 (also known as DCT)^3,4^. The mature melanosomes are then transported along microtubules and actin filaments toward the plasma membrane^5^. Kinesin superfamily proteins, such as KIF5B, facilitate microtubule-based transport, while the RAB27A-MLPH-MYO5A complex is essential for actin-based transport within melanocytes^6,7^. The mature melanosomes are ultimately transferred to neighboring keratinocytes *via* dendritic extensions or filopodia tips, resulting in skin pigmentation. Small GTPases, such as CDC42 and RAB17, mediate dendritic growth and filopodia formation, whereas RAC1 promotes melanocyte membrane ruffling and lamellipodia formation^8–10^. In addition, RAB11B is involved in the exocytosis of melanin from melanocytes^11^.

The MC1R/cAMP/MITF signaling pathway plays a pivotal role in regulating melanin production and pigmentation. Upon UV exposure, keratinocytes secrete melanocyte-stimulating hormone (α-MSH), which binds to the MC1R receptor on melanocytes, leading to elevated levels of cyclic AMP (cAMP)^12^. Elevated cAMP levels promote the transcription of MITF, the master regulator of melanocyte function and melanin biosynthesis^13^. MITF activates the expression of genes involved in melanin synthesis (e.g., *TYR*, *TRP-1*, and *TRP-2*), melanosomal components (e.g., *PMEL*), and melanosome trafficking machinery (e.g., *CDC42*, *RAB17*, *RAC1*, and *RAB27A*), ultimately leading to increased melanin production and accumulation^9,13–15^.

Oxyresveratrol (2,3’,4,5’-tetrahydroxystilbene) is a naturally occurring stilbene derivative found in plants, particularly in the Moraceae family^16^. It has attracted attention for its simple chemical structure and diverse pharmacological properties, including antioxidant, anti-inflammatory, anti-melanogenic, and anticancer effects^16–19^. Previous studies have demonstrated the depigmenting effects of oxyresveratrol both in vitro and in vivo; however, the precise mechanisms underlying these effects remain unclear^20–22^. It’s reported that pterostilbene, a structural analogue of oxyresveratrol, inhibited melanocyte dendricity and melanosome transport *via* the cAMP/PKA/CREB pathway^23^. Furthermore, a recent study suggested that oxyresveratrol regulated melanosome transfer by modulating the expression of RAB27A, RAC1, and CDC42^24^. However, direct evidence for oxyresveratrol’s role in suppressing melanin transfer is still lacking. Despite these findings, the effects and mechanisms of oxyresveratrol on dendrite formation and melanosome transport remain largely unexplored.

This study aims to investigate the effects and mechanisms of oxyresveratrol on melanin production and transport. The inhibitory effect of oxyresveratrol on tyrosinase activity was assessed and compared to other compounds using recombinant human tyrosinase. Additionally, the impact of oxyresveratrol on melanogenesis and the MC1R/cAMP/MITF signaling pathway was evaluated in B16F10 melanoma cells. The effects of oxyresveratrol on melanocyte dendritic growth and melanosome transport were further explored in a co-culture model of B16F10 and HaCaT cells. This study provided evidence that oxyresveratrol inhibited melanin synthesis and transfer through modulating the MC1R/cAMP/MITF signaling pathway, offering valuable insights into its potential therapeutic applications for the treatment of pigment-related disorders.

## Results

### Oxyresveratrol inhibited the activity of human tyrosinase

**Fig. 1:**
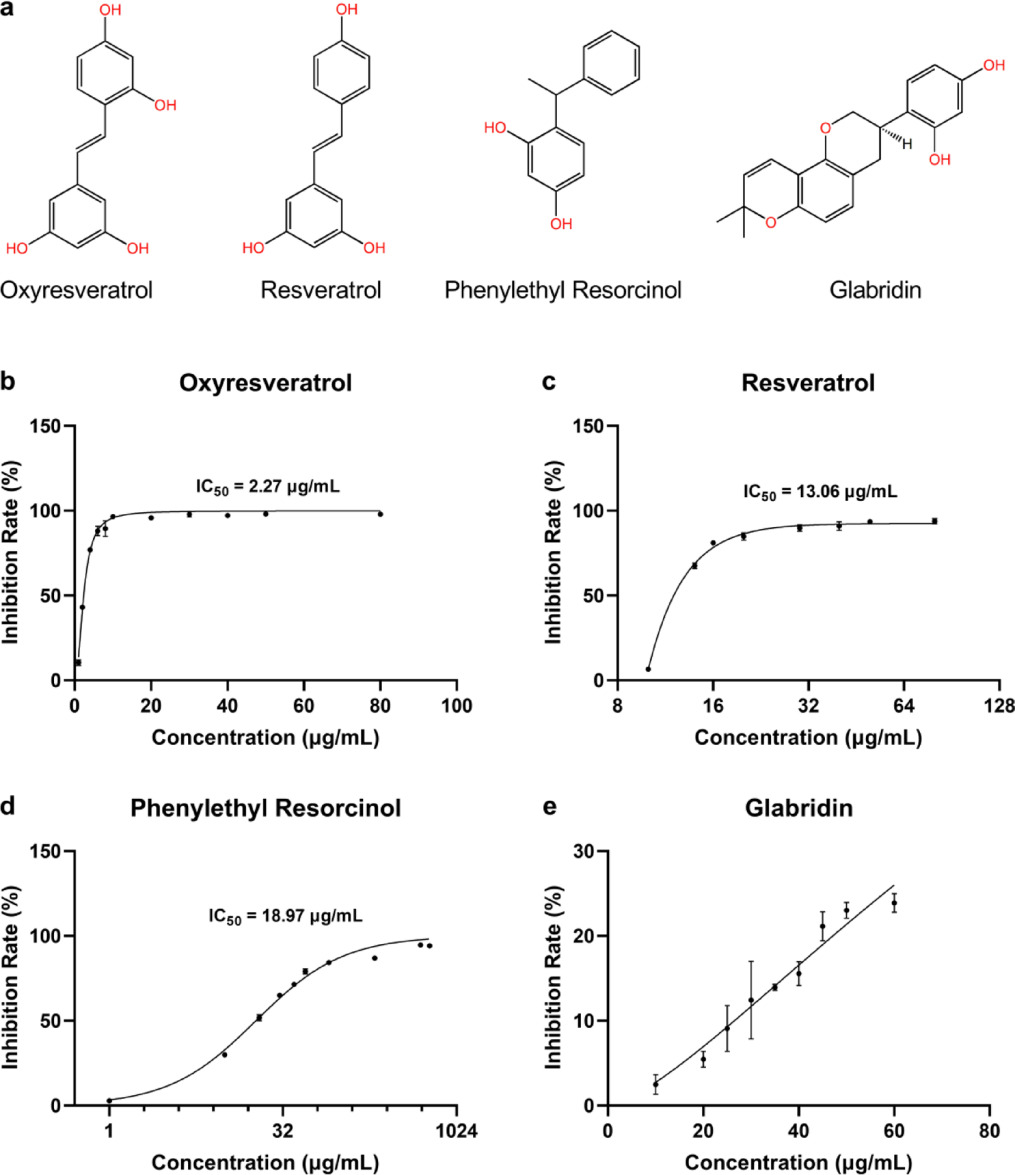
Chemical structure and inhibition effects of oxyresveratrol, resveratrol, phenylethyl resorcinol and glabridin on human tyrosinase activity. (**a**) Chemical structure of oxyresveratrol, resveratrol, phenylethyl resorcinol and glabridin. (**b**–**e**) Inhibition of human tyrosinase activity by oxyresveratrol, resveratrol, phenylethyl resorcinol, and glabridin in vitro, using *L*-DOPA as substrate. *N* = 3.

In this study, the ability of oxyresveratrol to inhibit human tyrosinase activity was evaluated and compared with that of other three rescorcinol-based compounds: glabridin, resveratrol, and phenylethyl resorcinol. The in vitro assay indicated that oxyresveratrol, resveratrol, and phenylethyl resorcinol effectively inhibited the oxidation of *L*-DOPA by human tyrosinase, with IC_50_ values of 2.27, 13.06, and 18.97 µg/mL, respectively (Table 1; Fig. 1). In contrast, the inhibitory effect of glabridin was unexpectedly low, with only a 23.88% inhibition rate at its saturation concentration of 60 µg/mL (Fig. 1e). In addition to IC_50_ values, the maximum inhibition rate of oxyresveratrol was up to 98.02%, surpassing that of resveratrol (93.78%) and phenylethyl resorcinol (94.65%), and was far more than glabridin (23.88%). These results indicated that oxyresveratrol is a potent inhibitor of human tyrosinase.

**Table 1:** Inhibitory effects of oxyresveratrol, resveratrol, phenylethyl resorcinol and glabridin to human tyrosinase activity.

No.	Compounds	IC_50_ (µg/mL)	Max inhibition rate (%)
1	Oxyresveratrol	2.27	98.02
2	Resveratrol	13.06	93.78
3	Phenylethyl resorcinol	18.97	94.65
4	Glabridin	> 60	≥ 23.88

### Oxyresveratrol inhibited melanin production in B16F10 cells

**Fig. 2:**
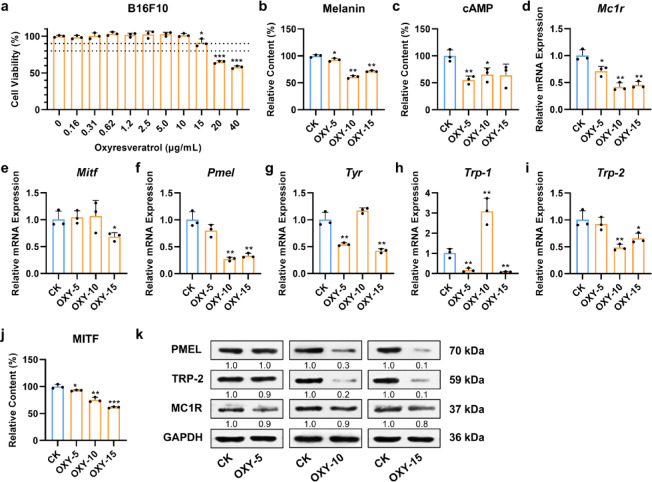
Oxyresveratrol inhibited melanin production through down-regulating the MC1R/cAMP/MITF signaling pathway in B16F10 cells. (**a**) The effect of oxyresveratrol on the viability of B16F10 cells was investigated by a CCK-8 assay. *N* = 3, Student’s *t* test, *** *P* < 0.001 vs. 0 µg/mL oxyresveratrol treatment. (**b**) The melanin content of B16F10 cells with or without oxyresveratrol treatment. CK, solvent control. OXY-5, 5 µg/mL oxyresveratrol group. OXY-10, 10 µg/mL oxyresveratrol group. OXY-15, 15 µg/mL oxyresveratrol group. Control cells (CK) were treated with the solvent (0.1% DMSO) equivalent to the highest concentration used in treatment groups. *N* = 3, Student’s *t* test, * *P* < 0.05, ** *P* < 0.01 vs. CK. (**c**) The cAMP levels of B16F10 cells with or without oxyresveratrol treatment were assessed by ELISA. (**d**–**i**) The effects of oxyresveratrol on the expression of six melanogenesis-related genes were evaluated by RT-qPCR in B16F10 cells. The mRNA expression levels of *Mc1r*, *Mitf*, *Pmel*, *Tyr*, *Trp-1*, and *Trp-2* were normalized to β-actin gene (*Actb*) levels. (**j**) The MITF protein levels of B16F10 cells with or without oxyresveratrol treatment were assessed by ELISA. (**k**) The effects of oxyresveratrol on the protein expression of PMEL, TRP-2, and MC1R in B16F10 cells. The protein levels of PMEL, TRP-2, and MC1R were normalized to GAPDH levels.

The impact of oxyresveratrol on melanogenesis was further confirmed in B16F10 cells. The results from the CCK-8 assay showed that B16F10 cells maintained over 90% viability after 24 h of incubation with oxyresveratrol concentrations ranging from 0 to 15 µg/mL, while a significant reduction in cell viability (< 80%) was observed at concentrations of 20 µg/mL and 40 µg/mL (*P* < 0.001 for both, Fig. 2a). Based on these results, concentrations of 5, 10, and 15 µg/mL were selected for subsequent experiments.

Next, melanin content in B16F10 cells was measured. Oxyresveratrol treatment resulted in a significant decrease in melanin production, with reductions of 7.2% (*P* = 0.050), 39.0% (*P* = 0.001), and 28.5% (*P* = 0.003) at concentrations of 5, 10, and 15 µg/mL, respectively, compared to the control (Fig. 2b). These results indicated that oxyresveratrol effectively suppressed melanin production in B16F10 cells.

### Oxyresveratrol down-regulated the MC1R/cAMP/MITF signaling pathway in B16F10 cells

To further elucidate the mechanism by which oxyresveratrol inhibits melanin production, we focused on the MC1R/cAMP/MITF signaling pathway. ELISA assays revealed that oxyresveratrol treatment significantly reduced cAMP levels in B16F10 cells by 44.7% (*P* = 0.004), 34.7% (*P* = 0.023), and 35.9% (*P* = 0.057) at concentrations of 5, 10, and 15 µg/mL, respectively, compared to the control (Fig. 2c). RT-qPCR analysis demonstrated considerable down-regulation of *Mc1r*, *Mitf*, and MITF-targeted genes, including *Pmel*, *Tyr*, *Trp-1*, and *Trp-2*, following oxyresveratrol treatment (Fig. 2d-i). The mRNA expression patterns of *Mc1r*, *Pmel*, and *Trp-2* closely mirrored the changes in melanin content with oxyresveratrol treatment (Fig. 2d, f, i). Specifically, 10 µg/mL oxyresveratrol treatment resulted in the most significant reduction in both melanin content and gene expression (*P* < 0.01 for all), followed by a decrease at 15 µg/mL (*P* < 0.05 for all). In contrast, treatment with 5 µg/mL only caused a slight reduction in both melanin content and gene expression. A significant decrease in *Mitf* expression was observed only after treatment with 15 µg/mL oxyresveratrol, with a 31.7% reduction (*P* = 0.030) compared to the control (Fig. 2e). Interestingly, mRNA levels of *Tyr* and *Trp-1* were significantly reduced after 5 µg/mL and 15 µg/mL oxyresveratrol treatment (*P* < 0.01 for all), but increased following treatment with 10 µg/mL (Fig. 2g, h). This may be attributed to negative feedback regulation, which needs further investigation. ELISA assays further revealed a dose-dependent downregulation of MITF protein levels upon oxyresveratrol treatment, with significant decreases of 7.1% (*P* = 0.039), 24.8% (*P* = 0.002), and 38.5% (*P* < 0.001) at concentrations of 5, 10, and 15 µg/mL, respectively, compared to the control (Fig. 2j). Additionally, western blot analysis also revealed a dose-dependent reduction in PMEL and TRP-2 protein levels (Fig. 2k). While *Mc1r* mRNA levels were markedly downregulated, its protein expression showed only slight reductions across all three treatment groups. This discrepancy may arise from post-transcriptional regulation or delayed protein turnover, which warrants further investigation. Original gels are presented in Supplementary Fig. S1. These results suggested that oxyresveratrol inhibited melanin synthesis in B16F10 cells by down-regulating the MC1R/cAMP/MITF signaling pathway.

### Oxyresveratrol suppressed melanin transfer from B16F10 to HaCaT cells

**Fig. 3:**
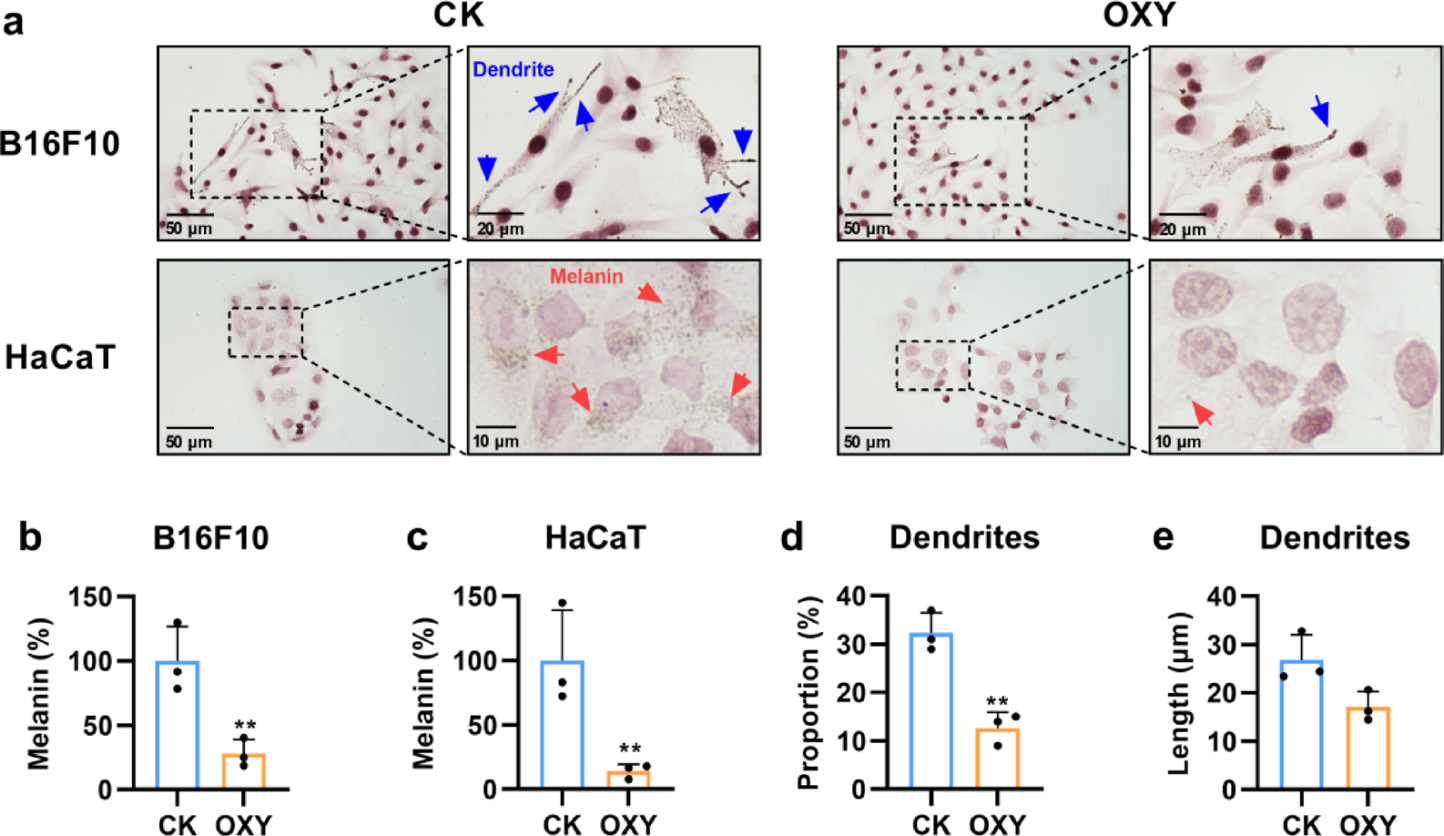
Oxyresveratrol suppressed melanin transfer and dendrite formation in the co-culture of B16F10 and HaCaT cells. (**a**) The micrograph of B16F10 and HaCaT cells with or without oxyresveratrol treatment. CK, solvent control. OXY, treatment with 10 µg/mL oxyresveratrol. Melanin and argyrophilic cell granules were stained in black, and the nucleus was stained in red. The dendrites of B16F10 cells were marked by blue arrows, while melanin in HaCaT cells was pointed by red arrows. (**b**,** c**) The melanin content of B16F10 and its transfer to HaCaT cells. (**d**) The proportion of dendritic B16F10 cells. (**e**) The average length of B16F10 dendrites. *N* = 3, Student’s *t* test, * *P* < 0.05, ** *P* < 0.01, *** *P* < 0.001 vs. CK.

The effect of oxyresveratrol on melanin transfer was investigated using a co-culture system of B16F10 and HaCaT cells. The 10 µg/mL concentration of oxyresveratrol was selected because it showed the lowest melanin content and did not affect cell viability (Fig. 2). Fontana-Masson staining was employed to visualize melanin granules in B16F10 and HaCaT cells (Fig. 3a). Treatment with 10 µg/mL oxyresveratrol resulted in significant reductions in melanin content both within B16F10 cells and in the transferred melanin to HaCaT cells, with decreases of 72.0% (*P* = 0.006) and 85.9% (*P* = 0.010), respectively (Fig. 3b–c). Notably, the reduction in melanin content in HaCaT cells was more pronounced than in the single-cultured B16F10 cells (Figs. 2b and 3c). These findings suggested that oxyresveratrol inhibited the transfer of melanin from B16F10 cells to HaCaT cells.

### Oxyresveratrol disturbed dendrite formation in B16F10 cells

Microscopic analysis further revealed that oxyresveratrol reduced the proportion of dendritic melanocytes and the average length of their dendrites by 60.8% (*P* = 0.001) and 23.2% (*P* = 0.111), respectively, compared to the control group (Fig. 3d, e). These results suggested that oxyresveratrol disturbed melanocyte dendrite formation.

**Fig. 4:**
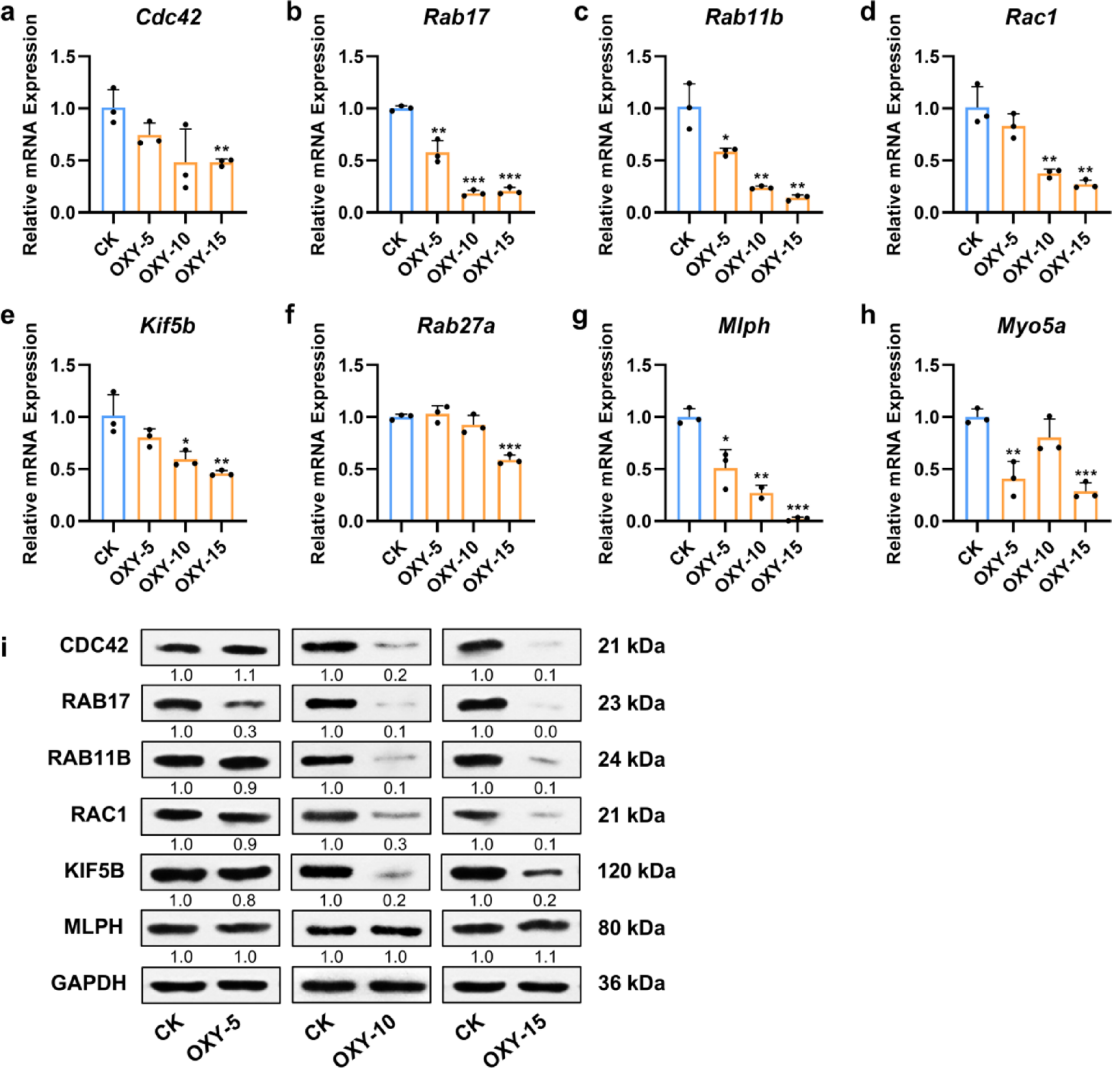
Oxyresveratrol inhibited the expression of small GTPases and kinesin involved in dendrite formation and melanosome transport. (**a**–**h**) The effects of oxyresveratrol on the expression of eight genes associated with dendrite development and melanosome transport in B16F10 cells. OXY-5, 5 µg/mL oxyresveratrol group. OXY-10, 10 µg/mL oxyresveratrol group. OXY-15, 15 µg/mL oxyresveratrol group. The mRNA expression levels of *Cdc42*, *Rab17*, *Rab11b*, *Rac1*, *Kif5b*, *Rab27a*, *Mlph*, and *Myo5a* were normalized to β-actin gene (*Actb*) levels. *N* = 3, Student’s *t* test, * *P* < 0.05, ** *P* < 0.01, *** *P* < 0.001 vs. CK. (**i**) The effects of oxyresveratrol on the protein expression of CDC42, RAB17, RAB11B, RAC1, KIF5B, and MLPH in B16F10 cells. The levels of these proteins were normalized to GAPDH levels.

**Fig. 5:**
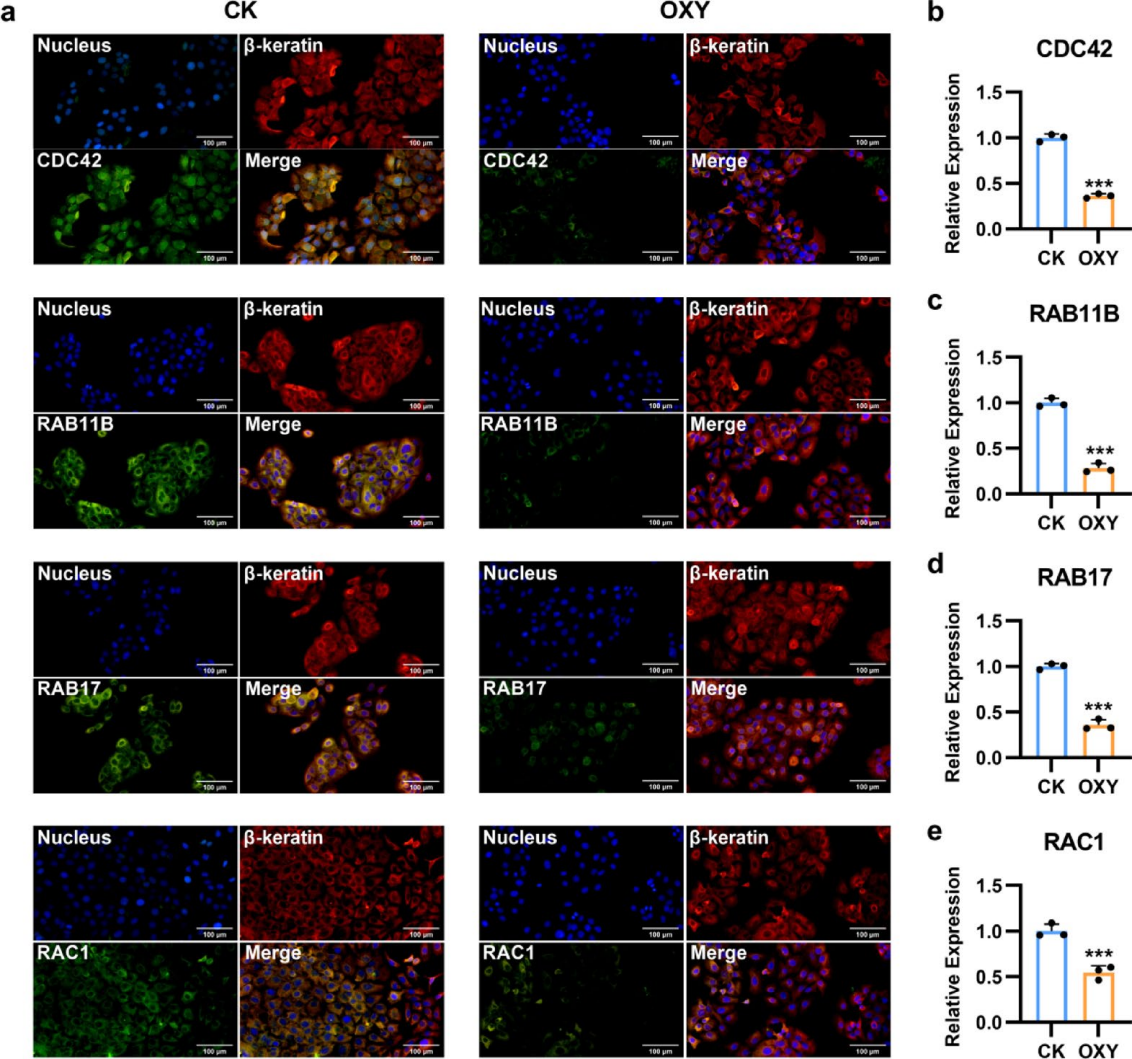
Oxyresveratrol did not alter the cellular localization but reduced the expression of CDC42, RAB11B, RAB17, and RAC1 in B16F10 cells. (**a**) The cellular localization of CDC42, RAB11B, RAB17 and RAC1 in B16F10 cells was detected by immunofluorescence. CK, solvent control. OXY, treatment with 10 µg/mL oxyresveratrol. The nucleus, keratin and corresponding proteins were labeled in blue, red and green, respectively. (**b**–**e**) The fluorescence level of CDC42, RAB11B, RAB17, and RAC1 in B16F10 cells. *N* = 3, Student’s *t* test, *** *P* < 0.001 vs. CK.

### Oxyresveratrol inhibited the expression of small GTPases and kinesin involved in dendrite formation and melanosome transport

Further investigation using RT-qPCR demonstrated that oxyresveratrol down-regulated the expression of genes associated with dendrite development and melanosome transport, encoding GTPases CDC42, RAB17, RAB11B, and RAC1, motor protein KIF5B, and the transport-related tripartite complex RAB27A-MLPH-MYO5A (Fig. 4a–h).

The gene expression patterns of *Cdc42* and *Rab17* in B16F10 cells treated with oxyresveratrol were consistent with those of *Mc1r*. Specifically, oxyresveratrol reduced the mRNA levels of *Cdc42* by 25.6% (*P* = 0.090), 51.9% (*P* = 0.065), and 51.9% (*P* = 0.006) at concentrations of 5, 10, and 15 µg/mL, respectively, compared to the control (Fig. 4a). Significant reductions in *Rab17* mRNA expression were observed following treatment with 5, 10, and 15 µg/mL oxyresveratrol, with decreases of 42.3% (*P* = 0.003), 81.5% (*P* < 0.001), and 79.4% (*P* < 0.001), respectively (Fig. 4b).

Oxyresveratrol treatment also resulted in a dose-dependent decrease in the mRNA levels of *Rab11b*, *Rac1*, *Kif5b*, *Rab27a*, and *Mlph* (Fig. 4c–g). In the 5 µg/mL oxyresveratrol group, no significant reduction was observed in the expression of *Rab11b*, *Rac1*, *Kif5b*, and *Rab27a*, but *Mlph* expression decreased by 49.0% (*P* = 0.012). In the 10 µg/mL group, significant decreases in the mRNA levels of *Rab11b*, *Rac1*, *Kif5b*, and *Mlph* were observed, with reductions of 76.2% (*P* = 0.004), 62.3% (*P* = 0.005), 40.6% (*P* = 0.029), and 72.9% (*P* = 0.002), respectively. In the 15 µg/mL group, significant decreases in the mRNA levels of *Rab11b*, *Rac1*, *Kif5b*, *Rab27a*, and *Mlph* were observed, with reductions of 85.7% (*P* = 0.002), 73.1% (*P* = 0.003), 54.0% (*P* = 0.009), 41.1% (*P* < 0.001), and 97.9% (*P* < 0.001), respectively.

Additionally, the gene expression pattern of *Myo5a* was similar to that of *Tyr*. Oxyresveratrol treatment resulted in a significant decrease in *Myo5a* expression, with reductions of 59.2% (*P* = 0.005), 19.6% (*P* = 0.147), and 71.3% (*P* < 0.001) at concentrations of 5, 10, and 15 µg/mL, respectively, compared to the control (Fig. 4h).

At the protein level, the expression of CDC42, RAB17, RAB11B, RAC1, and KIF5B was reduced dose-dependent following oxyresveratrol treatment, whereas MLPH levels showed no substantial change (Fig. 4i). Original gels are presented in Supplementary Fig. S1.

Immunofluorescence assays showed that RAB11B, RAB17, and RAC1 were localized within the cytoplasm of B16F10 cells, while CDC42 was present in both the nucleus and cytoplasm (Fig. 5a). Although oxyresveratrol treatment did not alter the cellular localization of these GTPases, it significantly reduced the fluorescence intensity of CDC42, RAB11B, RAB17, and RAC1 by 63.7%, 71.9%, 64.4%, and 45.6% (*P* < 0.001 for all), respectively, compared to the control group (Fig. 5b–e). These results suggested that oxyresveratrol suppressed melanin transfer by inhibiting melanocyte dendrite formation and melanosome transport, potentially through down-regulating the expression of CDC42, RAB17, RAB11B, RAC1, and KIF5B.

## Discussion

Oxyresveratrol, a natural derivative of resveratrol, has drawn attention for its various biological activities, including antioxidant, anti-inflammatory, anti-melanogenic, and anticancer effects^16–19^. Previous studies have demonstrated that oxyresveratrol suppressed melanin synthesis through inhibiting tyrosinase activity and down-regulating the expression of MITF, TRP-1, and TRP-2 in B16 cells^21^. A clinical study involving 60 female volunteers showed that a 0.25% heartwood extract of *Artocarpus lakoocha* Roxb., which contains 80% oxyresveratrol, significantly reduced melanin content in the upper arm after 4 weeks of application, with no reported adverse effects, suggesting the safety and depigmentating effect of oxyresveratrol in clinics^22^. Besides, previous studies suggested that oxyresveratrol may inhibit melanosome transport^23,24^. However, the precise effects and mechanisms of oxyresveratrol in regulating melanin synthesis and transfer remain unclear.

Tyrosinase is the key enzyme for melanin production within melanocytes and serves as a core target for treating pigment-related disorders. Although many tyrosinase inhibitors have been identified and used in the treatment of hyperpigmentation, many lack clinical efficacy because they were tested using mushroom tyrosinase^25^. The molecular structure of mushroom tyrosinase differs significantly from that of human tyrosinase, and the inhibitory efficacy of a compound on mushroom and human tyrosinase could vary by hundreds or even thousands of times^25^. Therefore, it’s necessary to reevaluate and compare the inhibitory effects of tyrosinase inhibitors using human tyrosinase. Various resorcinol-based compounds exhibit inhibitory effects on tyrosinase activity, such as phenylethyl resorcinol, glabridin, resveratrol, and its derivative, oxyresveratrol. A molecular docking study suggested the potential inhibitory activity of glabridin against human tyrosinase^26^while oxyresveratrol, resveratrol, and phenylethyl resorcinol were identified as potent inhibitors of human tyrosinase, with IC_50_ values of 0.7 µM (0.17 µg/mL), 2.6 µM (0.59 µg/mL), and 131 µM (28 µg/mL), respectively^20,25^. In this study, the inhibitory abilities of these four resorcinol-based compounds on tyrosinase activity were reevaluated and compared using recombinant human tyrosinase. As shown in Table 1; Fig. 1, oxyresveratrol, resveratrol, and phenylethyl resorcinol exhibited strong inhibition of human tyrosinase activity in vitro with IC_50_ values of 2.27 µg/mL, 13.06 µg/mL, and 18.97 µg/mL, respectively. In contrast, glabridin exhibited unexpectedly low inhibitory activity, with only a 23.88% inhibition rate at the saturation concentration of 60 µg/mL (Fig. 1e). Although glabridin had previously been identified as the most potent inhibitor of mushroom tyrosinase in nature^27^our findings suggested that its inhibitory effect on human tyrosinase activity was not commensurately strong. These results positioned oxyresveratrol as a potent inhibitor of human tyrosinase, surpassing the inhibitory effects of resveratrol, phenylethyl resorcinol, and glabridin.

In addition to tyrosinase, the MC1R/cAMP/MITF signaling pathway plays a pivotal role in melanin production and pigmentation^12–15^. In this pathway, oxyresveratrol has been proven to inhibit melanin production through down-regulating the expression of MITF, TYR, TRP-1, and TRP-2^21^. However, its effects on cAMP and MC1R levels have not been thoroughly investigated. In this study, considerable decreases in melanin content, cAMP levels, and the gene expression of *Mc1r*, *Mitf*, and MITF-targeted genes (*Pmel*, *Tyr*, *Trp-1*, and *Trp-2*) were observed in B16F10 cells treated with oxyresveratrol (Fig. 2b–i). ELISA and western blot assays further confirmed that the protein levels of MITF, MC1R, PMEL, and TRP-2 were reduced following treatment with 15 µg/mL oxyresveratrol (Fig. 2j–k). Notably, while 24-hour oxyresveratrol induced a modest reduction in *Mitf* mRNA levels only at the concentration of 15 µg/mL (Fig. 2e), it caused a marked decrease in MITF protein (Fig. 2j), suggesting that *Mitf* mRNA levels might have been transiently suppressed at earlier stages, followed by recovery through potential feedback mechanisms. Similarly, *Mc1r* gene expression was significantly suppressed (Fig. 2d), whereas MC1R protein levels exhibited a slight decline (Fig. 2k), likely due to the delayed turnover of pre-existing MC1R protein. This temporal dissociation between transcriptional and translational changes aligns with prior evidence that MITF activates *Mc1r* transcription^28^suggesting that the observed suppression of *Mc1r* transcription may be secondary to MITF inhibition rather than a direct effect of oxyresveratrol. Although our current data are limited to a single timepoint, future time-course studies tracking the MC1R/cAMP/MITF signaling will clarify these transient effects. Additionally, although cAMP reduction at 15 µg/mL OXY was not statistically significant (*P*= 0.057), the trend supports pathway modulation (Fig. 2c). Variability in cAMP assays or transient signaling effects may underlie this observation. These results confirmed the inhibitory effect of oxyresveratrol on melanogenesis and provided evidence for oxyresveratrol on regulating cAMP and MC1R levels. Together, these results suggest that oxyresveratrol inhibits melanin production by down-regulating the MC1R/cAMP/MITF signaling pathway.

The MC1R/cAMP/MITF signaling pathway regulates not only melanin synthesis but also melanosome trafficking machinery. It’s reported that melanocyte-stimulating hormone (α-MSH), an MC1R agonist, induced melanosome transport by promoting melanocyte filopodia formation^12,29^. The MITF regulates a series of genes involved in melanosome trafficking, including *CDC42*, *RAB17*, *RAC1*, and *RAB27A*^9,13–15^. Furthermore, a recent study demonstrated that oxyresveratrol treatment resulted in reduction of RAB27A, RAC1, and CDC42 in human melanocyte^24^. This observation led us to investigate whether oxyresveratrol has inhibitory effects on melanocyte dendrite formation and melanin transfer. In this study, oxyresveratrol was found to significantly reduce both the number and length of melanocyte dendrites, thereby decreasing melanin transfer from B16F10 cells to HaCaT cells (Fig. 3). To explore the underlying mechanisms, we focus on genes related to melanosome transport, particularly those downstream of the MC1R/cAMP/MITF pathway. Combined with the results of qRT-PCR, western blot, and immunofluorescence assays, this study found that oxyresveratrol significantly down-regulated the protein levels of CDC42, RAB17, RAC1, RAB11B, and KIF5B as well as the gene expression of *Rab27a*, *Mlph*, and *Myo5a* in B16F10 cells (Figs. 4 and 5). The KIF5B motor protein and the RAB27A-MLPH-MYO5A tripartite complex are essential for mature melanosome transport within melanocytes^6,7^while CDC42, RAB17, RAC1, and RAB11B mediate melanocyte dendritic development and the exocytosis of melanin^8–10^. Collectively, these results indicated that oxyresveratrol disturbed melanocyte dendrite formation and suppressed melanosome transport by modulating the expression of small GTPases (CDC42,RAB17, RAC1, and RAB11B), the kinesin KIF5B, and potentially the RAB27A-MLPH-MYO5A complex.

These findings suggested that oxyresveratrol could be a promising agent for treating melanoma and hyperpigmentation. Melanoma, a highly aggressive form of skin cancer originating from melanocytes, accounts for a relatively small percentage of skin cancer cases but is responsible for the majority of skin cancer-related deaths due to its rapid progression and metastasis^30^. The metastasis of melanoma, which involves local invasion, intravasation into blood or lymphatic vessels, survival in circulation, extravasation into distant tissues, and the establishment of secondary tumors, is the leading cause of mortality in advanced melanoma patients^31,32^. Each of these steps is regulated by complex signaling pathways that influence cell migration, adhesion, and the ability to adapt to new microenvironments^32^. In particular, the activation of small GTPases such as CDC42, RAB17, RAC1, and RAB27A, which regulate cytoskeletal dynamics, is thought to contribute to melanoma progression and metastasis^33–38^. In this study, the expression of these small GTPases was significantly reduced in B16F10 melanoma cells following oxyresveratrol treatment (Figs. 4 and 5), suggesting the potential of oxyresveratrol on inhibiting melanoma invasion and metastasis. These results are consistent with previous findings that oxyresveratrol inhibited the migration of human melanoma A375 cells, providing new insights into its molecular mechanisms^39^. In addition, overproduction and abnormal distribution of melanin can lead to skin hyperpigmentation, which not only affects appearance but also causes psychological distress, including anxiety disorders in some patients^40,41^. Topical treatments for hyperpigmentation typically focus on inhibiting melanin synthesis and transfer, with common agents including hydroquinone, corticosteroids, and niacinamide^42–44^. In the present study, oxyresveratrol demonstrated potent inhibitory effects on both melanogenesis and melanosome transport, suggesting its significant potential as a treatment for hyperpigmentation.

This study has several limitations: (1) The tyrosinase inhibitory assays were conducted using recombinant human tyrosinase, which may exhibit different activity compared to that in vivo; (2) Only two cell lines, the mouse melanoma B16F10 cells and human immortalized keratinocyte HaCaT cells, were used in this study, and the B16F10 cells are not of human origin; (3) Further experiments are required to explore whether oxyresveratrol affects melanin uptake by HaCaT cells, which is in the research plans. (4) Additional evidence at the protein level is necessary to confirm whether oxyresveratrol modulates the expression of the RAB27A-MLPH-MYO5A complex. (5) Clinical studies are required to validate the efficacy of oxyresveratrol.

In conclusion, the present study provided evidence for the effects and mechanisms of oxyresveratrol in regulating melanin synthesis and transfer. Specifically, oxyresveratrol treatment resulted in a reduction in the expression level of MC1R, cAMP, MITF, and MITF-targeted genes in B16F10 cells, indicating that oxyresveratrol down-regulated the MC1R/cAMP/MITF signaling pathway. Furthermore, it suppressed melanin transfer through inhibiting dendrite development and melanosome transport *via* regulating small GTPases (CDC42, RAB17, RAC1, and RAB11B) and kinesin (KIF5B). These findings highlight the inhibitory effect of oxyresveratrol on both melanin synthesis and transfer, proposing a potential treatment for melanoma and hyperpigmentation.

## Materials and methods

### Materials

Oxyresveratrol (CAS: 29700-22-9, purity ≥ 99%) was provided by Naturalis Srl (Italy). Resveratrol (CAS: 501-36-0, purity ≥ 98%) was obtained from Royal DSM (Netherlands). Phenylethyl resorcinol (CAS: 85-27-8, M866076, purity ≥ 98%) was purchased from MACKLIN (China). Glabridin (CAS: 59870-68-7, SG8480, purity ≥ 98%) and PBS buffer were purchased from Solarbio (China). Recombinant human tyrosinase (TP321797L) was obtained from OriGene (USA). *L*-DOPA (CAS: 59-92-7, S20191, purity ≥ 99%) was purchased from Shanghai Yuanye Bio-Technology (China). Dulbecco’s modified Eagle medium (DMEM, 2105341) was purchased from Gibco (USA). The 6-well transwell plates were purchased from Corning (China). Cell Counting Kit-8 (CCK-8, C6005M) was purchased from UElandy (China). M-MLV Reverse Transcriptase (M1705) and GoTaq^®^ qPCR Master Mix (A6002) were purchased from Promega (USA). The RIPA lysis buffer (G2002), BCA protein assay kit (G2026), and PMSF (G2008) were obtained from Servicebio (China). The mouse cyclic adenosine monophosphate (cAMP) ELISA kit (JL13362-96T) was purchased from JONLNBIO (China). The Microphthalmia-associated Transcription Factor (MITF) ELISA kit (ml037252) was purchased from mlbio (China). For western blot assay, antibodies CDC42 (10155-1-AP, 1:1000), RAC1 (24072-1-AP, 1:1000), MLPH (10338-1-AP, 1:1000), KIF5B (21632-1-AP, 1:1000), RAB11B (67780-1-Ig, 1:1000) and TRP-2 (13095-1-AP, 1:1000) were purchased from proteintech (China), RAB17 (TA350622S, 1:1000) and MC1R (TA321478S, 1:1000) were purchased from OriGene (USA), PMEL/gp100 (ab137078, 1:1000) was purchased from Abcam (UK), and GAPDH (KC-5G5, 1:10000) was purchased from Aksomics (China). Secondary antibodies Goat Anti-Rabbit IgG(H + L), Mouse/Human ads-HRP (4050-05, 1:20000) and Rabbit Anti-Mouse IgG(H + L)-HRP (6170-05, 1:10000) were obtained from SouthernBiotech (USA). For immunofluorescence, antibodies RAB11B (28498-1-AP, 1:1000) and Pan-keratin (26411-1-AP, 1:200 ~ 1:800) were purchased from proteintech (China), while RAB17 (DF9815, 1:1000) was purchased from Affinity (USA). DAPI staining solution (BS097) was purchased from Biosharp (China), and the mounting medium (S2100) was obtained from Solarbio (China). Other reagents and kits for western blot and immunofluorescence were purchased from Servicebio (China). The Masson-Fontana Melanin Stain Kit (G2032) was purchased from Solarbio (China). Other reagents were of the highest quality obtainable.

### Human tyrosinase Inhibition assay

The anti-tyrosinase activities of oxyresveratrol, resveratrol, phenylethyl resorcinol and glabridin were assessed using the method of the previous study with slight modifications^45^. Briefly, the solution containing human tyrosinase (final concentration of 25 U/mL), different concentrations of tested compounds, and PBS buffer was added into a 96-well plate. The mixture was incubated for 5 min at 37 °C. Then, *L*-DOPA (final concentration of 0.5 mM) was added and incubated at 37 °C for 10 min. Finally, the absorbance at 492 nm was measured on a microplate reader, SkyHigh (Thermo Fisher, USA). The inhibition rate was calculated according to the following formula:

$${\text{Inhibition}}\;{\text{rate}} = \left( {{\text{A}}2 - {\text{A}}1} \right)/\left( {{\text{B}}2 - {\text{B}}1} \right)*100\%$$

where A1 and A2 denote the absorbance of the reaction mixture with tested compounds before or after incubation, and B1 and B2 denote that without tested compounds before or after incubation, respectively.

### Cells and cell culture

The mouse B16F10 melanoma cells (CRL-6475) were obtained from ATCC (USA), and the human immortalized keratinocyte (HaCaT) cells were obtained from CLS (Germany). B16F10 and HaCaT cells were cultured in high-glucose DMEM medium supplemented with 10% FBS, 100 U/mL penicillin, and 100 µg/mL streptomycin at 37 °C with 5% CO_2_. Cells were split every 2–3 days at a ratio of 1:3 using 0.25% trypsin-EDTA. HaCaT cells used in experiments were between passages 15–20.

### Cell viability

The effect of oxyresveratrol on B16F10 cell viability was investigated by the CCK-8 assay as previously described^45^. Briefly, B16F10 cells were seeded in 96-well plates at a density of 1.5 × 10^4^ cells per well for 24 h, and then treated with varying concentrations of oxyresveratrol for 48 h. After treatment, 10 µL of CCK-8 reagent was added to each well and incubated for 2 h at 37 °C. The absorbance was measured at 450 nm.

### Measurement of melanin content

B16F10 cells were seeded into 6-well plates at a density of 5 × 10^5^ cells per well. After 24 h of incubation, cells were treated with different concentrations of oxyresveratrol for an additional 24 h. After treatment, cells were lysed by 1 mol/L NaOH containing 10% DMSO at 80 °C for 2 h. Then the supernatant was collected, and the melanin content was measured at 405 nm and normalized to the control. The assay was performed in triplicates (three independent wells) for each treatment group.

### Enzyme-linked immunosorbent assay (ELISA)

B16F10 cells were seeded in 96-well plates at a density of 1.5 × 10^4^ cells per well for 24 h, and then treated with oxyresveratrol for 24 h. The supernatant of cell lysate was used to assess the levels of cAMP and MITF protein using ELISA assay kits, following the manufacturer’s instructions.

### Real-time quantitative polymerase chain reaction (RT-qPCR)

The total RNA from cultured cells was extracted with TRIzol reagent and reverse-transcribed to cDNA using the M-MLV Reverse Transcriptase (Promega, USA). The primers for genes were synthesized by Sangon (China) (Table 2). RT-qPCR was then performed using the GoTaq^®^ qPCR Master Mix (Promega, USA) and the CFX96 Touch Real-Time PCR Detection System (Bio-Rad, Hercules, USA). Relative mRNA levels were calculated using the ^2−ΔΔ^Ct method after normalization to the level of β-actin.

**Table 2:** Primer sequences.

Gene	Forward (5′–3′)	Reverse (5′–3′)
*Actb*	CATTGCTGACAGGATGCAGAAGG	TGCTGGAAGGTGGACAGTGAGG
*Mc1r*	GAAAGGTGGCTCAGGGACATA	AGCCACCAACACCAGCAAGAT
*Mitf*	AGTGAGTGCCCAGGTATGAACA	GAGACGGGTAACGTATTTGCCA
*Pmel*	GAAAGGTGGCTCAGGGACATA	AGCCACCAACACCAGCAAGAT
*Tyr*	GAAGCGAGTCTTGATTAG	AGGTCGTAGATGTTGATA
*Trp-1*	GCTTGGAGGTCCGTGTATTTG	GGTTTGTCCTCCCGTTCCATT
*Trp-2*	CCTCAGGAACTGGCACCCA	CCCAGGATTCCAATGACCACT
*Cdc42*	AGATAACTCACCACTGTCCAAAGA	AGCACTCCACATACTTGACAGCC
*Rab17*	GGCTGCCTCTTTGTCCATTC	CTTCTGGGAGCCATTTGACA
*Rab11b*	TACCGTGCCATTACCTCTGC	GCCCACCAGCATGATGACAA
*Rac1*	ACACCACTGTCCCAATACTCCT	AGCACTCCAGGTATTTGACAGC
*Kif5b*	CAGGAACGGCTAAGGGTGG	GGTCTCCTCCAAACCCTTCAA
*Rab27a*	CCAGAGGGCAGTGAAAGAGG	TCCAGGAGCATCTCAATCGC
*Mlph*	GCTATGCCTGGAACAGACGAT	TGACATTCGCTTGTTCAACTCC
*Myo5a*	CATTGTGGAGCAGGCGAAG	TCATAGCGCTCCTCCAGGC

### Co-culture system of B16F10 and HaCaT cells

The co-culture system of B16F10 and HaCaT cells was established using the method of the previous study with slight modifications^46^. Briefly, B16F10 and HaCaT cells were respectively seeded at a density of 1 × 10^4^ cells/cm^2^ at the either side of permeable inserts in 6-well transwell plates. The B16F10 cells were cultured in the lower wells and the HaCaT cells were in the upper wells. The 0.4 μm holes in the insert membrane allows the transfer of melanosomes from B16F10 cells to HaCaT cells. The cells were incubated for 24 h and then treated with different concentrations of oxyresveratrol in fresh medium for 24 h. Finally, the two types of cells were respectively harvested for further analysis.

### Fontana-Masson staining

Fontana-Masson staining was employed to visualize melanin granules in the co-culture system. B16F10 and HaCaT cells were fixed with 4% paraformaldehyde for 30 min, and incubated with Fontana ammoniacal silver solution in the dark at room temperature for 24 h. Subsequently, cells were treated with hypo solution and neutral red each for 5 min. After dehydration in graded ethanol (95% for 5 min, absolute ethanol for 5 min), slides were air-dried at 37 °C and mounted with neutral gum. Melanin granules appeared black, while nuclei were stained red. The images were captured using a microscope (Mshot MDX10, China) and analyzed with Image-Pro Plus 6.0 software. Dendrite length was measured by tracing individual dendrites from the cell body to the tip. At least 100 cells per group were analyzed across three independent experiments.

### Western blot

Soluble proteins were obtained from lytic cells using RIPA lysis buffer, and the concentrations were measured by BCA protein assay kit. The crude proteins (20 µg per lane) were separated by 10% SDS-PAGE and transferred onto a PVDF membrane. The membrane was blocked with 5% non-fat skim milk for 2 h at 4 °C and washed three times with TBST buffer. It was then incubated with the appropriate primary antibodies overnight at room temperature. The following day, the membranes were treated with appropriate secondary antibodies for 1 h. All bound antibodies were visualized and analyzed using the chemiluminescence imaging system (CLINX 6300, China).

### Immunofluorescence staining

The cells were fixed with 4% paraformaldehyde and permeabilized with 0.5% TritonX-100. The tablet was then incubated with an endogenous peroxidase blocker for 15 min in the dark. Then, cells were blocked with PBS buffer containing 5% BSA for 30 min, and incubated with the corresponding antibodies: CDC42, RAB11B, RAB17, and RAC1 at 4 °C overnight. Subsequently, cells were incubated with the corresponding secondary antibodies for 30 min at room temperature. Nuclei were then stained with 1 µg/mL DAPI for 10 min in the dark at room temperature, and anti-fade mounting medium was applied to the slides. Finally, images were scanned by a fluorescent microscope (Mshot CKX53, China). Fluorescence intensity was quantified using ImageJ. Three fields per slide (≥ 30 cells per field) were analyzed across three independent experiments.

### Statistical analysis

The data, presented as mean values with standard deviations (mean ± SD), were analyzed statistically using IBM SPSS Statistics version 26. The Student’s *t* test was employed to assess differences between groups. The significance thresholds were designated as follows: * *P* < 0.05, ** *P* < 0.01, and *** *P* < 0.001. Figures were created using GraphPad Prism version 8.0.

## Electronic supplementary material

Below is the link to the electronic supplementary material.

Supplementary Material 1

## Data Availability

The data that support the findings of this study are available from the corresponding author, Jianhua Zhang, upon reasonable request.
