# Supplementary material for: Oxyresveratrol suppressed melanogenesis, dendrite formation, and melanosome transport in melanocytes via regulation of the MC1R/cAMP/MITF pathway
Source: Sci Rep. 2025 Jul 1;15:20400. doi: 10.1038/s41598-025-05248-x (PMC12218520; doi:10.1038/s41598-025-05248-x)
Supplement: Supplementary file 1 — Supplementary Material 1 [file 41598_2025_5248_MOESM1_ESM.pdf]

Marker 1 2 3 4 5 6 (1, 3, 5: Control group; 2, 4, 6: Oxyresveratrol treatment)

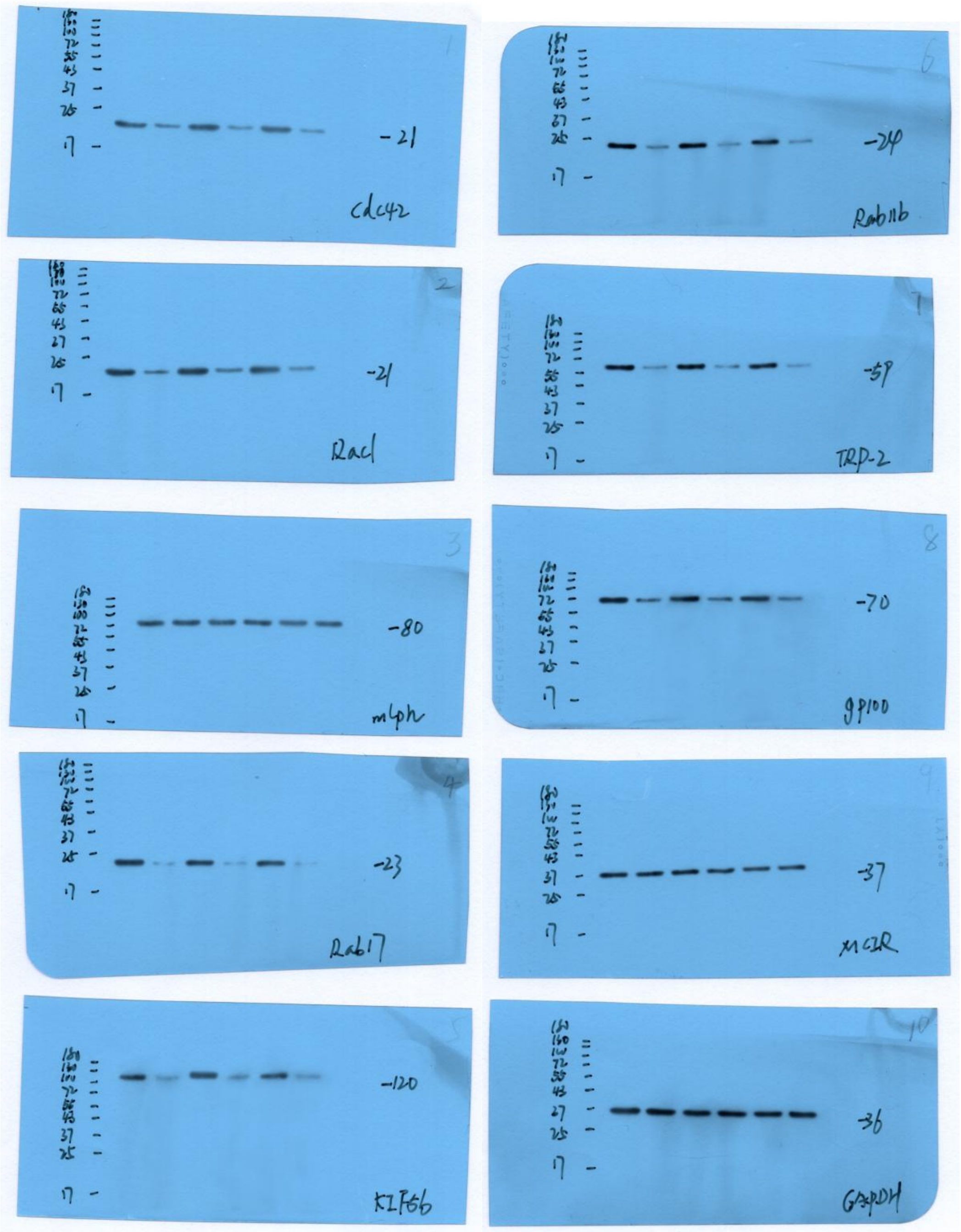

Figure S1. Original WB gel images of proteins involved in melanin synthesis and transfer after oxyresveratrol treatment. The images display Western blot (WB) results for the following proteins, from left to right and top to bottom: CDC42, RAB11B, RAC1, TRP-2, MLPH, PMEL (gp100), RAB17, MC1R, KIF5B, and GAPDH. GAPDH is used as the loading control, and protein levels are normalized to the GAPDH levels. The leftmost lane of each gel contains the molecular weight marker, followed by lanes 1, 3, and 5 representing the control group from three independent experiments, and lanes 2, 4, and 6 showing the corresponding 10 μg/mL oxyresveratrol treatment group. The molecular weight of the target proteins is indicated to the

right of the respective bands.
